# Effect of a calcitonin gene-related peptide-binding L-RNA aptamer on neuronal activity in the rat spinal trigeminal nucleus

**DOI:** 10.1186/s10194-018-0832-8

**Published:** 2018-01-15

**Authors:** Michael J. M. Fischer, Jakob Schmidt, Stanislav Koulchitsky, Sven Klussmann, Axel Vater, Karl Messlinger

**Affiliations:** 1 Institute of Physiology and Pathophysiology, University of Erlangen-Nürnberg, Universitätstrasse 17, D-91054 Erlangen, Germany; 2 Center for Physiology and Pharmacology, Medical University of Vienna, Vienna, Austria; 3 Department of Pharmacology, University of Liège, Liège, Belgium; 4 Aptarion Biotech, Berlin, Germany

**Keywords:** Migraine, Headache, Meningeal nociception

## Abstract

**Background:**

Calcitonin gene-related peptide (CGRP) plays a major role in the pathogenesis of migraine and other primary headaches. Spinal trigeminal neurons integrate nociceptive afferent input from trigeminal tissues including intracranial afferents, and their activity is thought to reflect facial pain and headache in man. CGRP receptor inhibitors and anti-CGRP antibodies have been demonstrated to be therapeutically effective in migraine. In parallel, CGRP receptor inhibition has been shown to lower spinal trigeminal neuron activity in animal models of meningeal nociception.

**Methods:**

In a rat model of meningeal nociception, single cell activity of neurons in the spinal trigeminal nucleus with meningeal afferent input was recorded to test a further pharmacological approach, scavenging CGRP with a CGRP-binding l-RNA oligonucleotide, the l-aptamer NOX-C89. Cumulative ascending doses of NOX-C89 were intravenously infused.

**Results:**

Spontaneous activity of spinal trigeminal neurons did not change after 0.05 mg/kg NOX-C89, however, after additional infusion of 0.5 mg/kg and 5 mg/kg NOX-C89, spontaneous activity was dose-dependently reduced. Identical doses of a control l-aptamer had no effect. This pharmacological effect of NOX-C89 was observed 10–25 min after infusion, but no difference was detected in the period 0–5 min. For comparison, the previously investigated CGRP receptor antagonist olcegepant had reduced activity within 5 min after infusion. Alongside the reduced spontaneous activity, after infusion of NOX-C89 the heat-induced neuronal activity was abolished.

**Conclusions:**

Scavenging CGRP by mirror-image RNA aptamers provides further evidence that this approach can be used to control spinal trigeminal activity.

## Background

The neuropeptide calcitonin gene-related peptide (CGRP), found in a subset of polymodal nociceptive afferents innervating intracranial tissues, is considered an important endogenous mediator in the generation of headaches. This notion is based on the observation that infusion of CGRP in migraineurs can trigger a migraine attack [1, 2] and, more importantly, inhibition of the CGRP system has been proven beneficial in migraine patients, whereby different ways of interfering with the CGRP system appear to be effective: Inhibition of CGRP release by triptans, CGRP receptor inhibition by gepants and scavenging CGRP or blocking its receptor by monoclonal antibodies.

### CGRP receptor antagonists

The CGRP receptor antagonists olcegepant (BIBN4096BS) and telcagepant (MK-0974) independently showed efficacy in phase III studies for the acute treatment of migraine [3–5]. However, olcegepant was clinically not further pursued, because of poor oral bioavailability, and telcagepant revealed some liver toxicity indicated by elevated transaminases when it was given daily to test its prophylactic use [6] or to prevent menstrual migraine [7]. This unclear toxic side-effect is reminiscent of preclinical experiments indicating a role of CGRP in liver inflammation and regeneration [8, 9], but might also be unrelated to the pharmacological effect. A third CGRP receptor antagonist, ubrogepant (MK-162), which has a different molecular structure, has successfully passed phase IIb [10] showing that this strategy has not been exhausted.

We hypothesized a central site of action for CGRP, and the existing evidence on this issue has been reviewed extensively [11–13]. Briefly, CGRP does not excite or sensitize meningeal nociceptors [14], but there are several lines of evidence suggesting a central site of action of CGRP, including the observation that CGRP receptor inhibition microiontophoretically injected at the recording site can reduce spinal trigeminal activity [15]. The central trigeminal activity was inhibited by systemically applied CGRP antagonists but not when the substance was applied to the dura mater; also effects on activity markers in the trigeminal ganglion were lacking when olcegepant was systemically applied [16–18]. Positron emission tomography of the CGRP antagonist telcagepant showed a rapid permeation of the blood–brain barrier and complete CGRP receptor occupancy at high concentrations. The receptor occupancy of an effective anti-migraine dose of telcagepant was lower than what is expected to be necessary [19], but useful levels of inhibition are rather based on general observations than any specific findings in headaches, and further depend on e.g. target desensitization, regional target composition or endogenous ligands [20].

### Scavenging strategies

Scavenging CGRP is another approach to migraine treatment. This requires large molecules with good tolerability, and some biologically occurring molecular classes seem to be well suited in this respect. One group is certainly antibodies, which are widely used therapeutically [21, 22]. Anti-CGRP antibodies, eptinezumab (ALD403 by Alder Biopharmaceuticals) galcanezumab (LY2951742 by Eli Lilly) and fremanezumab (TEV-48125 by Teva Pharmaceuticals), have a half-life of 21–48 days. The completed phase II trials for the prevention of migraine attacks in patients suffering from frequent migraine attacks showed promising results and the phase III trials are ongoing [23–26].

Another less explored option is chirally inverse l-oligonucleotides (l-aptamers, also known as Spiegelmers). RNA aptamers in the naturally occurring d-configuration that are enzymatically degraded by nucleases. In contrast, l-aptamers are stable in blood, resulting in a long half-life [27]. Such mirror-image (l-)aptamers have been developed to target gonadotropin releasing hormone, enterotoxin B, vasopressin, substance P, nociceptin, CCL2/MCP-1, amylin, ghrelin, hepcidin, CXCL12/SDF-1, C5a, sphingosine 1-phosphate and glucagon [27]. Three l-aptamers have passed clinical phase I and IIa trials. NOX-H94 for hepcidin completed a phase I trial (van Eijk et al. 2014), and NOX-E36 for CCL2 as well as NOX-A12 against CXCL12/SDF-1 have completed and published Phase IIa trials [28, 29]. Beyond stability, the rate of excretion is important for the plasma half-life. The latter can be prolonged by covalent coupling of polyethylene glycol moieties to these substances [30]. NOX-C89, a PEGylated variant of the first CGRP-binding l-aptamer, STAR-F12-Δ43–48 [31], that preferentially binds to mouse/rat αCGRP has been used to elucidate strain differences in rats regarding CGRP release and behavioral responses to noxious heat [32]. It was furthermore reported to reduce CGRP-dependent vasodilatation and blood flow in the exposed rat dura mater [33–35]. A newer anti-αCGRP l-aptamer NOX-L41, with equal affinity to mouse/rat αCGRP and an even higher affinity to human αCGRP, was shown to inhibit neurogenic plasma protein extravasation [36]. Before clinical validation, new strategies are tested in preclinical models [34, 37]. We chose to investigate neuronal firing in the trigeminal pathway in rats following our prior study, in which we examined the effect of the CGRP receptor antagonist olcegepant. Neuronal activity in the trigeminal system, particularly in the neurons receiving input from the cranial dura mater, is assumed to reflect headache. Therefore, the effects of an anti-CGRP l-aptamer on neuronal activity in the spinal trigeminal nucleus were tested. This approach has limited output at relatively high effort, but can directly asses the activity considered to underlie headaches. To this end experiments on cats and rats are established. Results do not point to a higher predictive value for human disease for one of these species; the current study used rats as in our prior work investigating olcegepant. The purpose of this study was to investigate the effect of anti-αCGRP l-RNA oligomers on the spontaneous and heat-evoked firing of spinal trigeminal neurons as a model of meningeal nociception.

## Methods

The experiments were performed in accordance with the ethical issues of the International Association for the Study of Pain ‘IASP Guidelines for the Use of Animals in Research’ and in compliance with the guidelines for the welfare of experimental animals of the Federal Republic of Germany and the European Commission (Directive 2010/63/EU). The experimental protocol was reviewed by an ethics committee and approved by the animal authority of the District Government of Mittelfranken or Unterfranken, Bavaria, Germany.

### Experimental procedures

Wistar rats were bred in house and held in group cages with a 12 h light/dark cycle at a temperature of 21 °C. Rats had a standard bedding material and access to pellet food and water ad libitum. Female rats were used for in house breeding, therefore male rats with a medium body weight of 365 g (range 300–480 g) were used for the experiments. Rats were drug naïve and had not undergone any procedures before the experiment. Details of the surgical and recording procedures were performed as described previously [16, 38]. Briefly, rats were anesthetized with either thiopental (Trapanal, Byk Gulden, Germany) as in prior investigations and with isoflurane once this was established. No difference in physiological parameters between the two regimes of anesthesia induction were apparent but the study was not powered to investigate this topic. For thiopental, an initial intraperitoneal dose of 120–150 mg/kg was injected, once the femoral vein was cannulated further adjustments were applied intravenously as indicated by nociceptive reflexes. Alternatively, the animals were placed into a closed box, which was supplemented by isoflurane 4% for rapid induction of anesthesia, continued with isoflurane 2.5% applied through a mask throughout the surgical procedures.

The right femoral artery was cannulated for monitoring arterial pressure and the right femoral vein to administer drugs. The animals were tracheotomized or intubated with an intravenous cannula (Vasuflo-T G14, Dispomed Witt, Germany). Atropine sulfate (B. Braun Melsungen AG, Melsungen, Germany, 0.5 mg/ml 1:10 with sodium chloride 0.9%) was injected subcutaneously to prevent salivation and muscle spasms. The animals were paralyzed with intravenous administration of gallamine triethiodide (40 mg/kg), and artificially ventilated with oxygen-enriched room air (about 30% *v*/v). Expiratory CO_2_ was monitored and maintained at 4.5–5%. Body temperature was maintained at 37–37.5 °C with a feedback-controlled homeothermic system (TKM 0902, Föhr Medical Instruments, Seeheim-Jugenheim, Germany). During the experiment, an isoflurane concentration of 1.5–2.0% was adequate to maintain a constant depth of anesthesia as required to suppress nociceptive reflexes or blood pressure changes evoked by noxious pinch stimuli of the hindpaw and the earlobe. Vital parameters (blood pressure, heart rate, expiratory CO_2_ level, and body temperature) were continuously recorded throughout the experiment. The experiments were terminated with an overdose of thiopental followed by exsanguination.

### Head surgery and electrophysiological recordings

The animal was placed in a stereotaxic frame with the head held in a fixed position by ear bars. The eyes were covered with dexpanthenol ointment (Bepanthen, Bayer Vital GmbH, Leverkusen, Germany) to prevent dehydration of the cornea. The skin overlying the skull was opened and the cranium exposed. Using a drill and fine forceps, a cranial window of 6 mm (rostro–caudal) and 4 mm (inferior–superior) was carefully cut into the left parietal bone to expose the dura mater. During surgery and throughout the experiment, the dura was protected from drying with isotonic saline. The neck muscles were divided along the animal’s midline, and the medullary brain stem was exposed by cutting a window into the atlanto-occipital ligament and the underlying dura mater. For recordings custom-made carbon fiber glass microelectrodes (impedance 1–2 MΩ) were used. Briefly, a gold-plated pin (diameter 1 mm) was connected to a silver wire of about 10 mm length, then a 10 μm thick carbon fiber was glued with conductive silver onto the wire. The fixed carbon fiber was sucked though a gentle airflow into a capillary (outer diameter 1.2 mm, Science Products) and the silver wire was inserted into the end of the capillary and fixed by a drop of two-component adhesive. The other end of the capillary was pulled out in a vertical pipette puller (Narishige, Tokyo, Japan), so that the carbon fiber was fused into the tip. The end of the fiber sticking out of the tip was shortened by etching with 0.1 M sulfuric acid under a microscope; ideally, the freely projecting carbon fiber tip is in the range of 20 μm. The microelectrode was inserted into the ipsilateral medulla and moved at steps of 2.5 μm using a custom-made motor-driven microstepper, controlled by a nano-stepper type B unit (Scientific Precision Instruments, Oppenheim, Germany).

Electric pulses of 0.5 ms duration and 10–12 V were delivered at an interval of 5 s to the exposed dura through bipolar gold electrodes with their rounded tips touching the dural surface. Neurons in the subnucleus caudalis of the spinal trigeminal nucleus (STN) with input from meningeal afferents were identified by their regular discharge in response to electrical stimulation and by their responses to mechanical probing of the dura with von Frey filaments (2.9–11.8 mN). Convergent afferent input from extracranial tissues was mapped by probing the ipsilateral cornea, temporal muscle, periosteum and facial skin with von Frey filaments and a fine blunt glass rod. Dural receptive fields were usually spot-like and located at or close to branches of the middle meningeal artery.

The extracellular signals were frequency filtered (high pass 100 Hz, low-pass 5 kHz), amplified, and digitized at a sampling rate of 20 kHz using a CED1402 controlled by spike2 software (Cambridge Electronic design, Cambridge, UK). Spike templates were generated off-line. The automatically generated templates in several runs with parameter adjustment were carefully considered to represent a single form, distinct from other signals. Thermally evoked action potentials were additionally used as templates to confirm the selected spikes. Thermally evoked action potentials were additionally used as templates to confirm the selected spikes. The position of recording sites was determined by measuring the distance caudal and lateral to the obex and by reading the depth of the microdrive. The center of the recordings was 2.4 mm caudal and 1.7 mm lateral of the obex; the average depth was 0.68 mm.

### Experimental protocols

Throughout the experiment, the rate of impulses per second was visualized and recorded for a control period of at least 20 min before application of substances. CGRP binding l-aptamer NOX-C89 and the control l-aptamer were synthesized and provided by NOXXON Pharma AG, Berlin. First, animals were dosed with 0.05 mg/kg, about 26 min later with 0.5 mg/kg and another 26 min later with 5 mg/kg. Substances were applied in a volume of 300 μl with an infusion pump at a rate of 120 μl/min over a period of 2.5 min. Consecutive experiments alternatingly tested NOX-C89 and the control l-aptamer. Baseline data between animals of experimental groups were not different.

Using a feedback-controlled vortex thermode [39], the surface temperature of the dura was kept at 32 °C. Thermal stimulation before and after every drug administration included two heat and one cold stimulation protocols as described previously [16]. The first heat stimulus consisted of a ramp with a slope of 0.1 °C/s from 32 °C to 44 °C. After 180 s at 32 °C the dura was heated to 36, 40 and 44 °C within 2 s in consecutive steps, each held for 30 s. After another 180 s at 32 °C, the temperature was decreased over 2 s to 26 and 20 °C, held for 30 s each, then returned to baseline. From each of 16 male Wistar rats, one neuron in the caudal subnucleus of the STN was recorded for the full protocol duration as described previously [40]. Sample size was based on the variance of neuronal activity observed in previous experiments.

### Substances

The CGRP-binding l-aptamer NOX-C89 consist of 42 nucleotides and has a molecular weight of 13.8 kDa, which is increased to a total of 54 kDa by addition of 40 kDa Y-shaped polyethylene glycol. NOX-C89 was initially dissolved as stock solution of 5 mg/ml in Dulbecco’s phosphate buffered saline with Ca^2+^/Mg^2+^ and further diluted in the same solution before intravenous infusion. The CGRP binding l-aptamer NOX-C89 was a slightly modified variant of STAR-F12-Δ43–48 [31] for improved synthesis. The nuclease resistant mirror-image (l-)RNA oligonucleotide has the sequence:

5´-GGACUGAUGG CGCGGUCCUA UUACGCCGAU AGGGUGAGGG GA-3′, which is PEGylated at its 5′ end via an aminohexyl-linker. The synthesis of this molecule has briefly been described [33]. In another series of control experiments, a non-functional control l-aptamer with the reverse nucleotide sequence of NOX-C89 was applied with the same experimental protocol.

### Data analysis

Results of two dependent samples were compared by Wilcoxon test for *n* < 10. Results from the two experimental groups and repetitive measurements were compared by ANOVA, followed by an LSD post hoc test. Normalized activity at single time-points was compared against 100% using a one-sample *t*-test. All tests were performed by the Statistica^®^ (StatSoft, USA) software package. All values are given as mean ± sem. Differences were considered significant at *p* < 0.05.

## Results

### General properties of units

All units had low threshold facial receptive fields predominantly in the ophthalmic region. The mean rate of spontaneous activity was 21 ± 7 imp/min (range 0.5–79 imp/min).

### Effects of NOX-C89 on the spontaneous activity

**Fig. 1:**
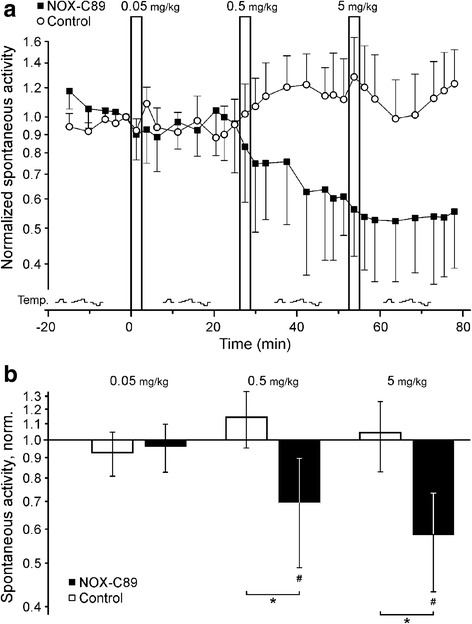
Anti-CGRP l-aptamer NOX-C89 reduces spontaneous neuronal activity. **a** Normalized activity of spinal trigeminal neurons. After infusion of NOX-C89, spontaneous activity was reduced. In contrast, infusion of the control l-aptamer did not alter neuronal activity. For both groups eight separate experiments were performed, symbols represent activity per 150 s. Note the longer intervals used for thermal testing, the heat and cold protocols are visualized at the bottom. **b** Twenty minutes after the infusion of NOX-C89 0.5 mg/kg and 5 mg/kg the spontaneous neuronal activity was reduced, both in comparison to baseline activity at the beginning of the experiment (7.5 min period before the first injection, ^#^*p* < 0.05) and to the control group (*p < 0.05). Data are mean ± SEM

After a control period of 20 min, intravenous infusion of the anti-CGRP l-aptamer NOX-C89 in three ascending doses caused a reduction in activity compared with control experiments (8 neurons in both groups, ANOVA, F_3,42_ = 3.6, *p* = 0.032, Fig. 1a). While NOX-C89 0.05 mg/kg had no significant effect, the infusion of 0.5 mg/kg and 5 mg/kg reduced neuronal activity in the period 10–25 min after infusion compared with experiments with the control l-aptamer (*p* = 0.042 and 0.039, LSD post hoc tests). Activity in the control group did not change significantly compared with the baseline before the first infusion. In the NOX-C89 group, neuronal activity was reduced to 70% after 0.5 mg/kg and to 54% after further 5 mg/kg compared with activity before the first infusion (*p* = 0.014, *p* = 0.039, single sample *t*-test vs 100%, Fig. 1b).

### Effects of thermal stimulation

**Fig. 2:**
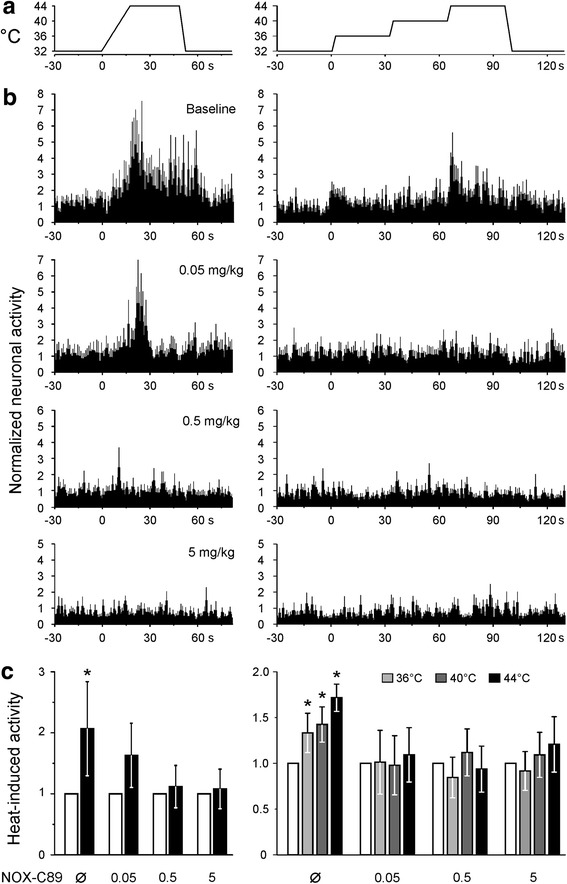
Heat evoked activity of units with afferent input from the exposed cranial dura mater recorded in the spinal trigeminal nucleus caudalis. **a** Heat sensitivity was obtained at baseline and after all infusions as visualized and at the time points shown in Fig. 1. The temperature at the dura was first ramped from 32 to 44 °C and held for 30 s. In a second protocol, the temperature was rapidly stepped from 32 to 36, 40 and 44 °C. **b** The response of eight spinal trigeminal neurons at baseline and after incremental additions of NOX-C89 0.05–5 mg/kg (mean ± sem) during the temperature protocols shown in panel A. **c** Average activity during the whole period of elevated temperature was increased above baseline. Similarly, all temperature steps increased neuronal activity. NOX-C89 infusion at all doses inhibited the neuronal response to thermal stimulation (*p < 0.05). Data are mean ± SEM

Increasing the local temperature on the dura mater in a ramp- or step-like mode caused an increase in neuronal activity (Fig. 2). During increased temperature (ramp from 32 °C to 44 °C within 18 s, 44 °C held for 30 s) the activity was 207 ± 77% compared with a 30 s control period before the stimulation (ANOVA, F_(1,32)_ = 4.8, *p* = 0.011, LSD post hoc test). The temperature steps caused a temperature-dependent increase in activity. The average activity at 36 °C was 133 ± 21%, at 40 °C 142 ± 19% and at 44 °C 172 ± 15% of the 30 s control period before the stimulation (ANOVA, F_(9,96)_ = 2.1, *p* = 0.015, 0.002 and 0.001, LSD post hoc tests). The thermal stimulation protocols were designed with different rates of temperature changes to detect phasic responses. Compared with the control period, evoked activity was 175 ± 18% in the first 10 s of the temperature increase and 133 ± 21% in the last 10 s of the temperature plateau. As previously reported, cooling of the dura did not change neuronal activity (data not shown).

### Effects of NOX-C89 on the activity evoked by thermal stimulation

Compared with initial thermal responses before NOX-C89 infusion, none of the thermal stimulations thereafter caused an increase in neuronal activity (temperature ramp: *p* = 0.18, 0.82 and 0.92, temperature steps *p* = 0.78, 0.81 and 0.70 for 0.05, 0.5 and 5 mg/kg, respectively, LSD post hoc tests).

## Discussion

Systemic infusion of an anti-CGRP l-aptamer slowly reduced spontaneous as well as heat-induced rat spinal trigeminal activity.

All recorded units had some spontaneous activity, and it cannot be excluded that experimental procedures contributed to the observed spontaneous activity. Experiments were performed in close adherence to the protocol used and published in a previous paper, investigating olcegepant [16]. Like the receptor antagonist, the CGRP-neutralizing l-aptamer NOX-C89 reduced spontaneous activity, however, only at a longer latency. In contrast to olcegepant, within 5 min after NOX-C89 application, no significant lowering of activity was observed. An explanation for this difference might be the molecular weight difference, i.e. the bulkier and charged NOX-C89 might have a slower uptake kinetic into the relevant compartment.

### Comparison to NOX-C89 effects in other studies

In a previous paper, a positive association of CGRP expression levels with heat sensitivity was demonstrated. The thermal sensitivity of mice expressing abundant CGRP was lowered after scavenging CGRP [32], which is consistent with our results. We have reported previously that NOX-C89 applied locally to the dura inhibited electrically evoked blood flow increases; a dose of 5 mg/kg reduced the evoked blood flow increases to 66% of the control [33]. This is similar to the 54% of remaining spinal trigeminal activity after a cumulative dose of 5.55 mg/kg in the present study, although it is unclear how well the vascular responses, which are frequently used as surrogates of the afferent activity due to their CGRP release, really correlate to neuronal activity [41, 42]. In a pressure myography of rat meningeal arteries in vitro, NOX-C89 reduced CGRP-induced dilatation when applied abluminally (IC_50_ 20 nM), but not luminally, indicating the requirement of sufficient CGRP concentration at the smooth muscle cells [34]. In vivo, NOX-C89 1 mg/kg caused about 80% inhibition of the CGRP-induced dilatation in rat dural and pial arteries compared with the response before the CGRP scavenger [35]. A minimum period of 30 min was between NOX-C89 infusion and the second CGRP-testing, which might be necessary for NOX-C89 distribution. This would be consistent with our finding of a slower onset of the NOX-C89 effect compared with olcegepant.

### Spinal trigeminal activity

Reduction of spontaneous activity and reduced thermal responsiveness were detected at different doses but occurred largely together. One could hypothesize that the activity of the spinal trigeminal neurons, which is at least partly driven by the activity of primary afferent neurons [43], is inhibited by targeting CGRP. This would explain the suppression of thermal responses, as there is no reason to assume that the peripheral terminals of the neurons lost their heat sensitivity. Considering that both NOX-C89 as well as olcegepant reduced the activity to about half in high doses, one might further hypothesize that the remainder is an independently driven spinal trigeminal neuron background activity.

### NOX-C89 site of action

CGRP secretion from primary afferents in the dorsal horn enhances thermal and mechanical nociceptive sensitivity [44, 45]. A simple assay to measure CGRP at this site has been developed [46, 47]. Considering the central site of action of CGRP receptor antagonists outlined in the introduction: Why would scavenging CGRP by a substance not necessarily penetrating the blood-brain–barrier be beneficial? A potential answer would be a site of action outside the blood-brain–barrier. In case the site of action is inside the blood–brain barrier, a low level of penetration might be sufficient or the tightness of the barrier is, at least temporary, altered. The latter is also a controversial issue, but there are studies in favor of this hypothesis, e.g. S100b plasma levels were associated with migraine attacks and are considered an index for blood–brain barrier alteration [48]. It should be mentioned that an effect of the anesthetic in this respect cannot be excluded. Extravasation of a fluorescently labeled version of the CGRP-neutralizing l-aptamer NOX-L41 (50 l-nucleotides conjugated to 40 kDa polyethylene glycol and Alexa488) in the dura mater started to become visible 5 min after administration and increased for about 2 h. Fluorescence was not detected in deeper cortical layers (below 55 μm), showing that NOX-L41-Alexa488 extravasates from dural but not from cortical vessels [36].

### Thermoresponses

A high amount of dural nociceptors, similar to the nociceptors throughout the body, are sensitive to thermal stimuli. The observed temperature responses are coding only rapid changes well; the required rate of change does not naturally occur at the dura. Therefore, the thermal stimuli should be considered an experimental means to assess the responsiveness of the respective afferents. We have demonstrated that these stimuli in turn affect meningeal perfusion [49]. The sensitization of primary meningeal afferents by inflammatory agents involved in migraine has been demonstrated [50]. An attempt to explain the thermal sensitivity is lowering of the thermal threshold below the local temperature by sensitizing inflammatory mediators. This mechanism has been demonstrated for peripheral neurons [51] but seems rather hypothetical given the minimal proportional response of dural afferent neurons.

### Comparison to other approaches targeting the CGRP system

The plasma half-life of l-RNA aptamers is about 1–2 days. This is much longer than the biologically occurring d-RNAs, and is not governed by degradation but by uptake into phagocytes, such as hepatic Kupffer cells. While there is no general rule for small molecules, the typical IgG antibody half-life in serum is around 20 days [52]. As mentioned above, eptinezumab galcanezumab and fremanezumab have a half-life above 20 days, which is appreciated if this successfully translates to long intervals between applications for prophylactic use. The molecular weight of NOX-C89 is about 54 kDa compared with the anti-CGRP antibodies with about 145 kDa. There are currently no efforts for a further clinical development of NOX-C89 or its follow-up candidate NOX-L41, but this might become more attractive in case the anti-CGRP antibodies are effective but need to be abandoned for unforeseen reasons.

## Conclusion

l-aptamer NOX-C89, belonging to a new chemical class of CGRP scavengers, was demonstrated to reduce spontaneous and thermally evoked activity in the spinal trigeminal nucleus neurons. Three l-aptamers have shown good safety and efficacy in clinical trials, and insights from these trials might help to progress the scavenging of CGRP as a therapeutic principle in migraine and possibly other headaches.

## Abbreviation

l-aptamer: Oligonucleotide in the unnatural L-configuration, also known as Spiegelmer, which has been designed to bind to a biological target of interest
